# *In vivo* burn scar assessment with speckle decorrelation and joint spectral and time domain optical coherence tomography

**DOI:** 10.1117/1.JBO.28.12.126001

**Published:** 2023-12-06

**Authors:** Qiang Wang, Peijun Gong, Hadi Afsharan, Chulmin Joo, Natalie Morellini, Mark Fear, Fiona Wood, Hao Ho, Dilusha Silva, Barry Cense

**Affiliations:** a The University of Western Australia, Optical+Biomedical Engineering Laboratory, Department of Electrical, Electronic and Computer Engineering, Perth, Western Australia, Australia; b Harry Perkins Institute of Medical Research, BRITElab, QEII Medical Centre, Nedlands, Western Australia, Australia; c The University of Western Australia, Centre for Medical Research, Perth, Western Australia, Australia; d The University of Western Australia, School of Engineering, Department of Electrical, Electronic & Computer Engineering, Perth, Western Australia, Australia; e Yonsei University, Department of Mechanical Engineering, Seoul, Republic of Korea; f The University of Western Australia, Burn Injury Research Unit, School of Biomedical Sciences, Perth, Western Australia, Australia; g Fiona Stanley Hospital, Fiona Wood Foundation, Murdoch, Western Australia, Australia; h Fiona Stanley Hospital, Burns Service of Western Australia, Western Australia Department of Health, Murdoch, Western Australia, Australia; i The University of Western Australia, Department of Electrical, Electronic and Computer Engineering, Microelectronics Research Group, Perth, Western Australia, Australia

**Keywords:** optical coherence tomography, angiography, flow measurement, skin, burn scar, burn scar assessment

## Abstract

**Significance:**

Post-burn scars and scar contractures present significant challenges in burn injury management, necessitating accurate evaluation of the wound healing process to prevent or minimize complications. Non-invasive and accurate assessment of burn scar vascularity can offer valuable insights for evaluations of wound healing. Optical coherence tomography (OCT) and OCT angiography (OCTA) are promising imaging techniques that may enhance patient-centered care and satisfaction by providing detailed analyses of the healing process.

**Aim:**

Our study investigates the capabilities of OCT and OCTA for acquiring information on blood vessels in burn scars and evaluates the feasibility of utilizing this information to assess burn scars.

**Approach:**

Healthy skin and neighboring scar data from nine burn patients were obtained using OCT and processed with speckle decorrelation, Doppler OCT, and an enhanced technique based on joint spectral and time domain OCT. These methods facilitated the assessment of vascular structure and blood flow velocity in both healthy skin and scar tissues. Analyzing these parameters allowed for objective comparisons between normal skin and burn scars.

**Results:**

Our study found that blood vessel distribution in burn scars significantly differs from that in healthy skin. Burn scars exhibit increased vascularization, featuring less uniformity and lacking the intricate branching network found in healthy tissue. Specifically, the density of the vessels in burn scars is 67% higher than in healthy tissue, while axial flow velocity in burn scar vessels is 25% faster than in healthy tissue.

**Conclusions:**

Our research demonstrates the feasibility of OCT and OCTA as burn scar assessment tools. By implementing these technologies, we can distinguish between scar and healthy tissue based on its vascular structure, providing evidence of their practicality in evaluating burn scar severity and progression.

## 1 Introduction

Burn injuries constitute a significant global health issue, responsible for ∼2.9  million hospitalizations and 176,000 deaths annually worldwide.^1^ The majority of such injuries are thermal, caused by fire and hot water, resulting in significant and often long-term physical and psychological implications.^2^ Non-fatal burn-related sequelae, such as scars, can cause altered skin appearance and function, with linked psychological impacts that can significantly decrease quality of life. Scarring can have multiple long-term impacts, including pruritus, pain, and restricted movement.^3^ Accurate assessment and measurement of scar severity is vital for monitoring the healing process and providing guidance on the appropriate treatment to improve the quality of life of patients.^4^

Currently several scar assessment methods are used, including the Vancouver Scar Scale (VSS), which quantifies vascularity, pliability, and height of a scar, and the Patient and Observer Scar Assessment Scale which assesses observer and patient opinions on different scar characteristics.^5^^,^^6^ These scales take into account factors, including height, pigmentation, thickness, pliability, and overall appearance of the scar.^7^^,^^8^ However, currently scar assessments are primarily subjective and limited in their reliability across different assessors and conditions.^9^ Recently, the use of ultrasound to measure scar thickness has also been adopted to provide a more objective measure, but this approach can be subject to operator-dependent variations, limiting its widespread acceptance.^10^ Therefore, objective measures of scars remain an important focus of research to support successful clinical intervention and evidence-based guidelines for scar minimization. Considering the evidence that burn scars demonstrate higher blood flow rates, which may contribute to accelerated healing,^11^ our study proposes a promising method to investigate variations in blood flow rates, alongside the assessment of microvessel count, structure, and distribution. This combined method offers a comprehensive strategy to better access burn scars. Since blood flow is inherently associated with a number of other scar properties, including color and maturation, this measure may be indicative of healing and provide an objective measure of outcome. Therefore, the aims of this study were to quantify the axial blood flow velocity, blood vessel density, and microstructure in both scar and healthy tissue, and evaluate their potential as scar assessment tools.

Optical coherence tomography angiography (OCTA) is a new imaging technology that offers three-dimensional (3D) visualization of the microvasculature.^12^^,^^13^ There are several variants of OCTA, each with its distinct capabilities. For example, Doppler optical coherence tomography (DOCT)^14^ and joint spectral and time domain optical coherence tomography (joint STdOCT)^15^ are capable of measuring flow velocity while speckle variance and speckle decorrelation^16^^,^^17^ can map the structure of the vasculature but determining flow velocity can be a challenge. In addition, the variable interscan time analysis (VISTA)^18^^,^^19^ method has been developed to measure relative flow speed but it is unable to provide measurements of absolute speed or flow direction.

DOCT, which utilizes the Doppler effect generated by moving blood cells, was the first OCTA variant capable of measuring blood flow speed in the axial direction. This method has demonstrated considerable potential for imaging blood flow in retinal tissue,^20^^,^^21^ cerebral tissue,^22^ and tumors.^23^^–^^25^ However, DOCT has several limitations. It demands high phase stability of the imaging hardware to accurately quantify slow flow velocities.^26^ In addition, as with other OCTA variants, DOCT is susceptible to shadow artifacts that can distort the flow signal in tissue beneath a vessel.^26^ Moreover, the data processing procedure of DOCT requires phase unwrapping to resolve π ambiguity, which refers to the inability to distinguish phase differences greater than π, and this can become complicated in the presence of phase noise and discontinuities.^27^

The speckle decorrelation technique processes the optical coherence tomography (OCT) intensity signal rather than the phase, making it well-suited for imaging vessel structures. In contrast to phase-based approaches such as DOCT, this method does not require phase information and thus is immune to phase noise. This method calculates the decorrelation value between sequential cross-sectional OCT images at the same location to identify regions of high decorrelation, which correspond to blood vessels. One key advantage of this method is its ability to generate 3D maps of microvessel structures using just two repeated B-scans collected from the same location. Furthermore, the long-time interval between B-scans allows the detection of slow flow, although this also makes it susceptible to artifacts resulting from motion between the OCT probe and the imaging sample that need to be minimized. Additionally, the size of the window used for decorrelation calculation may limit the effectiveness of this method in detecting small vessels.

The VISTA method is an extension of the decorrelation technique, which provides a measurement of the relative flow velocity of the blood. The VISTA method acquires multiple B-scans at a specific location in a short time and then performs decorrelation analysis on these scans at different time intervals to provide a measure of the changes in the decorrelation signal strength over time. Through this comparative analysis, the method derives information about the relative flow speed of blood vessels, offering the unique advantage of assessing flow without phase signals. The VISTA method is especially sensitive to low flow speeds, but requires more robust hardware for rapid acquisition of multiple B-scans. Despite the constraint, the VISTA method has the potential to provide relative flow dynamics, especially in scenarios where low flow speeds are of interest.

Joint STdOCT, a variant of OCTA, enables the measurement of blood flow velocity and was first introduced by Szkulmowski et al.^15^ Joint STdOCT utilizes two Fourier transformations applied simultaneously to spectra acquired at the same locations over time to determine flow information. Exhibiting a higher sensitivity for detecting velocity at low OCT signal-to-noise ratios (SNRs) compared to Doppler-based methods, joint STdOCT has proven to be a valuable tool in flow analysis. However, this method requires a time-series of data points, which constrains visualization to cross-sectional views instead of 3D images within a practical time frame.

Multiple papers have been published using OCT as a tool for scar assessment. For example, the work of Liew et al.^28^ quantified the vessel density in projection vessel images of scars, showing increased vessel density in scar areas compared to healthy skin. Similarly, Lu et al.^29^^,^^30^ extended their investigation to include blood vessel density, surface roughness, and optical attenuation coefficient (OAC) assessment by OCT and showed that scar regions tended to have smoother surfaces and a lower OAC compared to healthy skin. Deegan et al.^31^ investigated the effects of burns on vessel depth. It provided new insights into the impact of burn injuries on the vasculature. Notably, polarization-sensitive OCT, an extension of OCT, was also used to measure the birefringence of scar tissue and, interestingly, found that birefringence tended to be higher in scars than that of the healthy skin.^32^

In this paper, we offer a comprehensive explanation of flow velocity derivation through Fourier transformation, and introduce an improved and standardized processing procedure to improve image quality and extract diverse information. We refer to this processing method as “enhanced STdOCT.” This innovative approach goes beyond conventional methods by not only considering vessel density, depth and size but also quantifying and analyzing axial blood flow velocity and microstructure in both scar and healthy tissue. Our results show the application of this improved method in burn scar analysis, along with a comparison to DOCT and speckle decorrelation method, ultimately evaluating the feasibility of utilizing OCTA for burn scar assessment.

## 2 Method

### 2.1 Theory

The interferometric expression of the detected spectral signal of the A-scan, I(k), for a single moving scatterer acquired from a spectral-domain OCT scanner, can be formulized as

$$I(k)=\rho S(k)\{R_{r}+R_{s}+2\sqrt{R_{r}R_{s}}\;\cos[2nk(z_{r}-z_{s}-v_{z}t)]\},$$ (1)

where ρ denotes the detector responsivity, and S(k) represents the power spectral density function of the OCT light source. $R_{r}$ and $R_{s}$ denote the reflectivities of the reference mirror and scatterer in the sample, respectively. The distances from the beam splitter to the mirror and scatterer are indicated by $z_{r}$ and $z_{s}$, respectively.^26^^,^^33^ The refractive index of the sample is represented by n while k is the wavenumber. $v_{z}$ denotes the axial velocity of the moving scatterer, and t is the time variable (i.e., the scan is acquired at time t). Motion of a scatterer (i.e., $v_{Z}\neq 0$) leads to a phase shift in the interferogram.

The inverse Fourier transform of the backscattered light interferogram is a function of the sample depth.^34^ Performing an inverse Fourier transformation along k to the detected interferogram in Eq. (1), the depth-dependent signal I(z) of the sample can then be reconstructed as

$$I(z)=\rho \gamma (z)(R_{r}+R_{s})+2\rho \sqrt{R_{r}R_{s}}[\begin{array}{c} \gamma (z-v_{z}t)e^{-i2nk_{0}(z-v_{z}t)}\\ +\gamma (-z+v_{z}t)e^{i2nk_{0}(z-v_{z}t)} \end{array}],$$ (2)

where z represents the length difference between the reference mirror and sample reflector while $k_{0}$ denotes the wavenumber of the center frequency in vacuum. The Gaussian-shaped coherence function γ(z) is the inverse Fourier transformation of S(k), which can be characterized by coherence length $l_{c}$ as $\gamma (z)=\exp[-4\;\ln\;2{\left(z/l_{c}\right)}^{2}]$. The depth reflectivity I(z) can be decomposed into two terms, as shown in Eq. (2). The first term is the pathlength-independent offset which does not contain flow information. The second term is the conjugate cross-correlation component which is caused by the moving scatterers. Only the positive side of the second term will be kept as the negative side is the conjugate of the positive side.

To implement the enhanced STdOCT, a time-series signal expressed in Eq. (2) is acquired. Performing the Fourier transformation to the time-series signal in Eq. (2) along the time t, the resulting frequency function of the pixel at depth $z_{0}$ can be expressed as

$$I(f{)}_{z=z_{0}}=\rho \gamma (z_{0})(R_{r}+R_{s})F(1)+2\rho \sqrt{R_{r}R_{s}}F[\gamma (z_{0}-v_{z}t)e^{-i2nk_{0}(z_{0}-v_{z}t)}].$$ (3)

Equation (3) can be simplified as

$$I(f{)}_{z=z_{0}}=A_{z_{0}}\cdot \delta (f)+\frac{\rho l_{c}\sqrt{R_{r}R_{s}\pi }e^{i2\pi k_{0}z_{0}}}{v_{z}\sqrt{\ln\;2}}\cdot e^{-\frac{\pi^{2}l_{c}^{2}}{4\;\ln\;2v_{z}^{2}}{\left(f-\frac{k_{0}nv_{z}}{\pi }\right)}^{2}}\cdot e^{-\frac{i2\pi z_{0}}{v_{z}}(f-\frac{k_{0}nv_{z}}{\pi })},$$ (4)

where $A_{z_{0}}$ is a constant for each specific depth $z_{0}$ and δ(f) denotes the Dirac delta function. As $I{\left(f\right)}_{z=z_{0}}$ is a complex function, we take the modulus to obtain the amplitude of its spectrum

$$\begin{array}{c} |I(f{)}_{z=z_{0}}|=A_{z_{0}}\cdot \delta (f)+B_{z_{0},v_{z}}\cdot e^{-\frac{\pi^{2}l_{c}^{2}}{4\;\ln\;2v_{z}^{2}}{\left(f-\frac{k_{0}nv_{z}}{\pi }\right)}^{2}}, \end{array}$$ (5)

where $B_{z_{0},v_{z}}$ is a constant for each specific depth $z_{0}$ and velocity $v_{z}$. After the Fourier transform, the first term transforms into a Dirac delta function at 0 Hz multiplied by a constant ($A_{z_{0}}$). The second term is the velocity term, a Gaussian-shaped function. The frequency of the center of the Gaussian-shaped function $f_{\text{center}}$ is equal to $nk_{0}v_{z}/\pi$. Therefore, after $f_{\text{center}}$ is identified in the frequency spectrum, the velocity can be calculated as

$$v_{z}=\frac{\pi f_{\text{center}}}{nk_{0}}.$$ (6)

Equation (6) provides the first explanation how the center frequency and the axial velocity are related to each other.

### 2.2 Experimental Setup

OCT scans were acquired using an upgraded TELESTO II scanner (Thorlabs Inc., New Jersey, United States) to demonstrate the OCTA methods on healthy human skin and human burn scars, *in vivo*. This system has a center wavelength of 1300 nm with an axial and lateral resolution of 5.5 (in air) and 13  μm (as defined by the vendor), respectively. In addition, the system offers several A-scan rate options, ranging from 10 to 146 kHz, with a sensitivity of 109 dB at 10 kHz and 91 dB at 146 kHz (according to the vendor specifications).

A custom-made silicone flow phantom was fabricated by mixing Elastosil P7676A and P7676B fluid (Wacker Chemie AG, Germany) with titanium dioxide in a 3D-printed resin container. The container had 2 holes in the sidewalls to hold a small glass cylindrical capillary (outer diameter: 330  μm; inner diameter: 200  μm) to mimic a blood vessel. After curing, the capillary was embedded in the solid silicone matrix that mimicked the static tissue. The scattering properties of the silicone matrix were adjusted by tuning the ratio of titanium dioxide to Elastosil P7676A and P7676B, which was ∼1:250:250 in weight. The phantom had an attenuation coefficient that approximately matched the attenuation of healthy human skin.^11^^,^^35^ To mimic blood flow, the capillary was connected to a syringe filled with a polystyrene microsphere (diameter: 0.5  μm) aqueous suspension (Polysciences, Inc., Pennsylvania, United States). The syringe was connected to a pump (Fusion 200, Chemyx Inc., Texas, United States) to control the flow speed. The phantom was fixed on an angle-adjustable stage to allow easy adjustment of the flow angle relative to the OCT beam.

*In vivo* scans were collected from the arms or legs of 10 human subjects (including 1 healthy subject and 9 patients) with approval from the Human Research Ethics Committee of The University of Western Australia (RA/4/1/4131) and South Metropolitan Health Service Ethics Committee WA (RGS0000004980). Written informed and voluntary consent was obtained from all subjects prior to OCT imaging.

Two types of *in vivo* skin OCT scans were acquired from each patient. The first was a 3D scan with two repeated B-scans at each location across a field of view (FOV) of 6.0×3.6  mm or 6.0×4.5  mm. This 3D scan was processed with the conventional speckle decorrelation method,^16^^,^^28^^,^^36^ to generate a map of the microvasculature. The resulting 3D image facilitated the selection of vessel locations for subsequent 2D data acquisition. The second type of scan was a 2D scan, in which 1000 A-scan locations were scanned over 6 mm with 1000 repeated A-scans acquired at each A-scan location. This 2D scan was then processed using both DOCT and enhanced STdOCT methods. To mitigate the effects of bulk tissue motion during *in vivo* data acquisition, an imaging spacer was attached to the skin surface to couple the OCT probe and the skin.^37^ Ultrasound gel was applied to the skin surface for refractive index matching, which reduced the surface reflection and imaging artifacts.^38^ The dataset comprised 27 groups of scans, including 3D scans and 2D scans located at the scar, the boundary between the scar and healthy skin, and healthy skin located ∼10  cm away from the scar area. The acquisition of scans at the scar and healthy skin boundary was motivated by the aim of validating the potential to detect scar edges. This validation was performed by investigating whether the features of blood vessels, as observed in burn scars and healthy skin, could also be detected at the burn scar boundary. The burn types included electric shock, contact, laser (for wart treatment), and open flame burns, and were located on the arm or leg of the patients. The age of the patients ranged from 19 to 60 years.

Blood flow in microvessels with a diameter ranging from ∼10  μm to more than 500  μm has been reported to have flow speeds ranging from ∼100  μm/s to more than 10  mm/s.^39^ Considering this flow speed range, the OCT scanner operated at 10 kHz for all *in vivo* 2D scans, and the acquisition of one *in vivo* 2D scan took ∼109  s. The velocity measurement range by DOCT and enhanced STdOCT is −2.2 to +2.2  mm/s (assuming a refractive index of 1.5 for the blood),^40^ which generally matched the expected range of blood flow velocity in the axial direction.

### 2.3 Data Processing

The TELESTO OCT system acquired a spectrum for each A-scan, which was linearly resampled in k-space before performing a Fourier transformation to generate complex OCT depth signals for all scans in this study. Complex OCT signals from repeated A-scans were utilized to form a time-series signal at each depth. Zero-padding was applied to the time-series signal before the Fourier transformation to achieve higher resolution in the resulting frequency domain signal. A Gaussian window was then multiplied with the signal to minimize sidelobe impact during the Fourier transform.

In accordance with Eq. (5), a narrow Gaussian band-rejection filter (center frequency: 0 Hz; full width at half maximum: 3% of the full frequency range) was applied to eliminate the Dirac delta function at 0 Hz from the frequency spectrum. The non-flow velocity signal displays symmetry about the y-axis in both the positive and negative frequency spectra. By computing the difference between the positive and negative spectra, we can effectively attenuate the non-flow velocity signal, thereby increasing SNR of the flow signal.

Two techniques were evaluated to determine the center frequency of the Gaussian-shaped function that represents the flow in the processed frequency spectrum. The first technique was curve fitting, which involved fitting a Gaussian function to the filtered spectrum. Through this technique, the center frequency of the Gaussian-shaped function was identified. The processing time for 1 depth pixel was ∼0.11  s, totaling 110 s for one B-scan. The second method was peak finding, which directly located the maximum amplitude in the processed spectrum and considered its corresponding frequency as the center frequency. This method was faster, taking only ~0.10 s to process one B-scan. When applied to the same scans, both methods produced nearly identical results, with a difference in the measured frequency of <4%. The center frequency was then used to calculate the axial flow velocity according to Eq. (6) while the positive or negative frequency range indicated the flow direction.

**Fig. 1:**
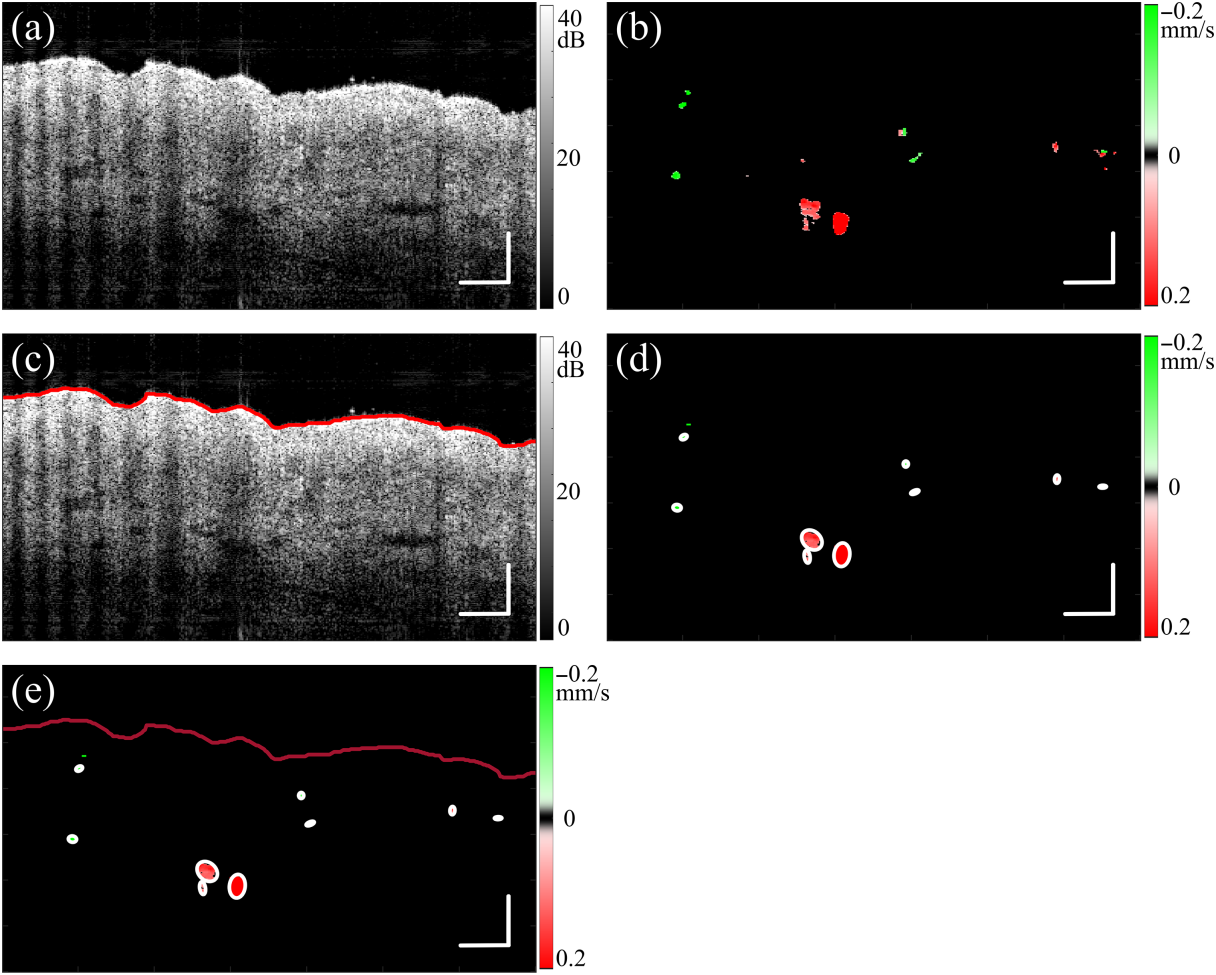
Flow chart illustrating the process of extracting blood vessel information. (a) OCT cross-sectional image; (b) OCTA image processed by enhanced STdOCT using the data as in panel (a); (c) OCT cross-sectional image with detected surface; (d) OCTA image with the blood vessels marked with ellipses; and (e) final result: skin surface, vessel locations, dimensions, and flow velocities. Scale bars: 200  μm.

When a comparison was made between enhanced STdOCT and DOCT methods, the axial flow velocity measured by DOCT was calculated using the equation $v(x,y,z)=\lambda_{0}\Delta \varphi (x,y,z)/4\pi nT$,^41^ where $\lambda_{0}$ is the center wavelength, Δφ(x,y,z) is the phase difference located at coordinate (x,y,z), and T is the time interval between A-scans. An example flow velocity image created using the enhanced STdOCT is shown in Fig. 1(b). Subsequently, an ellipse-fitting algorithm was applied to identify the boundaries of individual blood vessels in the diagram, as shown in Fig. 1(d). Each ellipse represents a cross-sectional view of a vessel, and the number of ellipses corresponds to the number of vessels within the imaged area. It can be assumed that blood vessels are shaped like round tubes. When an OCT image is taken, it captures a slice of the round tube, which appears as an ellipse when the slice is not perpendicular to the blood vessel. The minor axis of the ellipse (the shorter diameter) is equal in length to the diameter of the circle. By measuring the minor axis of the ellipse, the diameter of the blood vessel can be determined. In addition, by analyzing the OCT image in Fig. 1(a), the position of the skin surface can be determined based on the region of the signal with the highest change in intensity of OCT signal along the depth. The depth of each blood vessel can be determined as the distance between the fitted ellipse and skin surface. It is important to note that due to signal attenuation, only vessels located from the skin surface to a depth of 1 mm (in optical path length) will be counted and analyzed.

The speckle decorrelation for the *in vivo* 3D skin scans was calculated with a previously reported method^11^ with a window size of 3×3  pixels, corresponding to a depth and width of 11 and 18  μm, respectively. The decorrelation was further weighted by the corresponding original logarithmic OCT signal amplitude to remove the artificially high decorrelation signal created by the noise in the OCT signal. Prior to weighting, the logarithmic OCT signal was thresholded by an empirically chosen threshold of 10 dB. The vessels from the skin surface to a depth of 600  μm into the skin were then projected for visualization of the microvasculature using the weighted decorrelation.

## 3 Results

### 3.1 Phantom Results

**Fig. 2:**
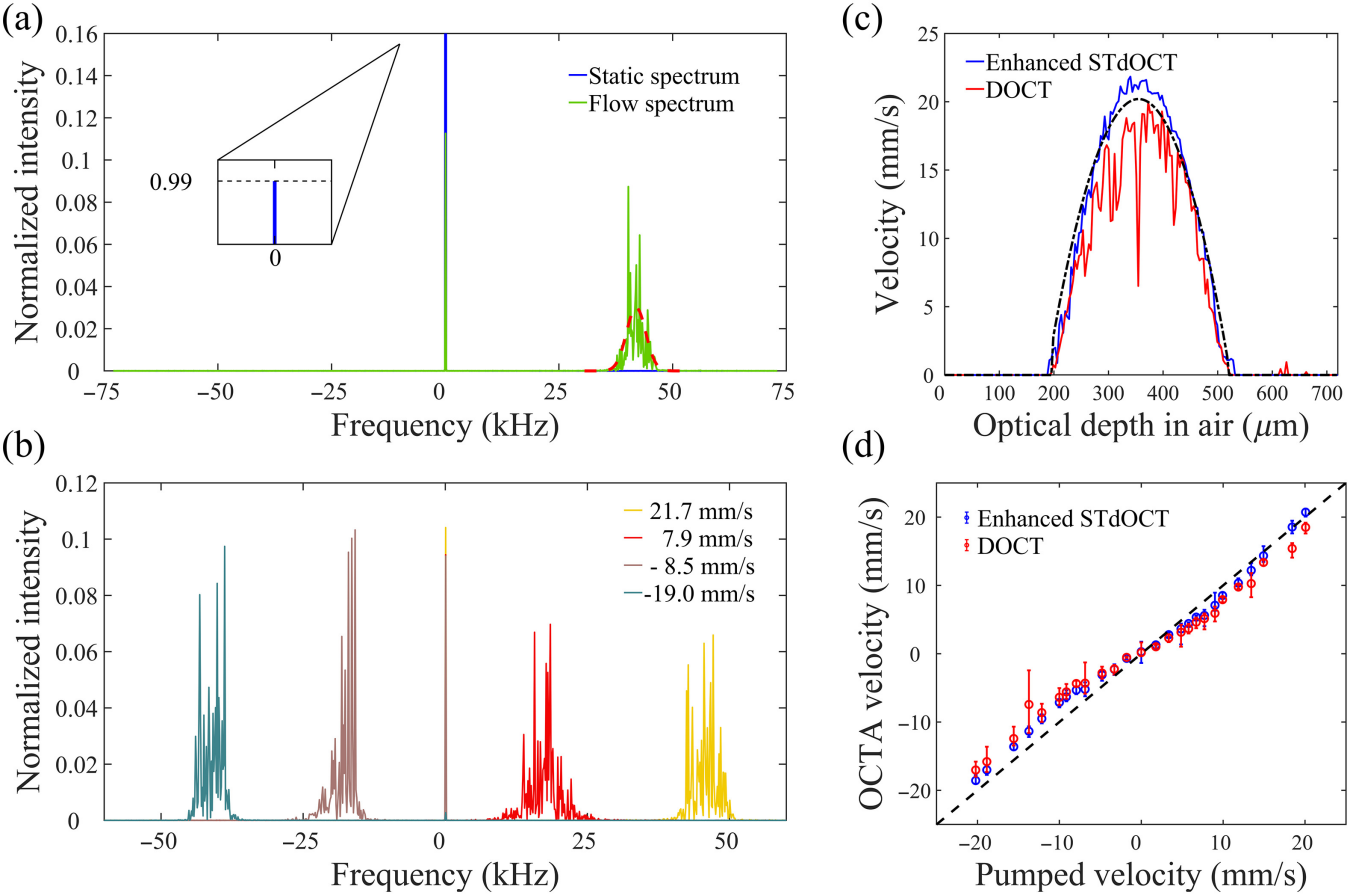
(a) Comparison of spectra from a static region (in blue) and flow region (in green) with a Gaussian fit to retrieve the peak induced by the flow centered at 42.6 kHz for the flow region (dashed red); (b) spectra from flow regions with different axial flow velocities; (c) velocity distribution along the depth across the capillary with enhanced STdOCT (blue), DOCT (red), and pump setting (black); and (d) velocity comparison between enhanced STdOCT (blue) and the DOCT (red) from all phantom scans. The dashed line indicates equal flow velocities for the OCT flow measurement (with enhanced STdOCT or DOCT) and pump setting. The error bar represents the standard deviation over 60 experiments.

Figure 2 shows the flow signal generated by applying enhanced STdOCT to OCT scans from the phantom. As explained in Sec. 2.1 [Eq. (5)], the spectrum of the time-series signal consists of a Dirac delta function and a Gaussian-shaped function for a flow region, in contrast to a static region, where only a single Dirac delta function exists. An example of such a comparison is shown in Fig. 2(a), taken from a static region outside the capillary (blue) and a flow region (135 deg and 20  mm/s) at the center of the capillary (green). The magnitude of the spectra across the detectable frequency range (−73 to 73 kHz) was normalized by dividing the original magnitude by the total power of the original time-series signal. The normalized magnitude in Fig. 2(a) shows the total signal power percentage [the vertical axis in Fig. 2(a) is cropped to display a range from 0 to 0.16]. Both spectra in Fig. 2(a) present a Dirac delta function with a width <0.5  kHz centered at 0 Hz, referred to as the static peak hereafter. The maximum magnitude of the static peak in the static region is more than 0.98 as shown in the inset of Fig. 2(a). In the flow region, a Gaussian-shaped function is observed corresponding to the flow, which is referred to as the flow peak. The blue and green spectra significantly overlap, except in the frequency range surrounding the static and flow peaks. The center of the flow peak from the flow region is located at ∼42.6  kHz, identified through curve fitting (dashed line). An axial flow velocity of 19.8  mm/s at the center of the capillary is obtained by Eq. (5), which agrees well with the set flow velocity. In the experiment, the average flow velocity of 20  mm/s was controlled by the pump while the refractive index of the aqueous suspension is assumed to be 1.4, and the flow is assumed to be laminar. There are two possible reasons why the measured velocity is slightly lower than the theoretical velocity. First, the measured velocity may represent the average velocity of a small area at the center of the capillary, as the resolution is not infinitely small. Alternatively, the actual flow angle could be slightly smaller than the set angle.

The ability to measure both the flow velocity and direction in the phantom scans is further demonstrated by the spectra in Fig. 2(b). These spectra were obtained from four different flow regions in the phantom with the same average flow speed but different flow angles. The two spectra (yellow and red), with the flow peaks located in the positive frequency range, are respectively from the center and side of the capillary. The pump controlled the average flow speed at 20.0  mm/s, and the flow angle was set at 135 deg. The laminar flow model was used to estimate the flow velocity of 21.5 and 8.0  mm/s for these 2 flow regions, based on the average flow speed and flow angle. Enhanced STdOCT demonstrated accurate measurements, with recorded flow velocities of 21.7 and 7.9  mm/s, as shown in Fig. 2(b).

Similarly, the two spectra (green and brown) with negative frequency peaks are from the center and side of the capillary, with an average flow speed of 20.0  mm/s and a flow angle of 45 deg. First, the flow peaks are in the negative or positive frequency range, respectively, when the flow angle is smaller than 90 deg (i.e., axial flow has the same direction as the OCT beam direction) or larger than 90 deg (i.e., axial flow direction is opposite to the OCT beam direction). This feature helps to resolve the flow direction, similar to DOCT. Second, a faster axial flow results in a larger shift of the flow peak relative to the static peak in Fig. 2(b). The center frequency of the flow peak is proportional to the set flow speed and helps to quantify the axial flow velocity based on Eq. (6).

Figure 2(c) shows a comparison of the flow velocities measured by enhanced STdOCT and DOCT. The graph in Fig. 2(c) shows the flow profile as a function of depth across the capillary. The dashed black curve is the theoretical laminar flow profile, estimated from an average speed of 20.0  mm/s with an angle of 135 deg in a straight tube with a circular cross-section.^42^ Both flow profiles determined by enhanced STdOCT and DOCT correspond well with a theoretical parabolic laminar flow profile. The enhanced STdOCT better matches the theoretical values, especially at the locations close to the center of the capillary. Measuring the velocities within the capillary at each depth from phantom scans with 4 average speeds (0, 5, 10, and 20  mm/s) and 5 flow angles (45 deg, 60 deg, 90 deg, 120 deg, and 135 deg), Fig. 2(d) provides a summary of the flow measurement accuracy compared to the velocities calculated from the pump setting. The velocities measured with enhanced STdOCT are more accurate and stable, as demonstrated by the smaller standard deviation in Fig. 2(d), compared to those measured with DOCT.

**Fig. 3:**
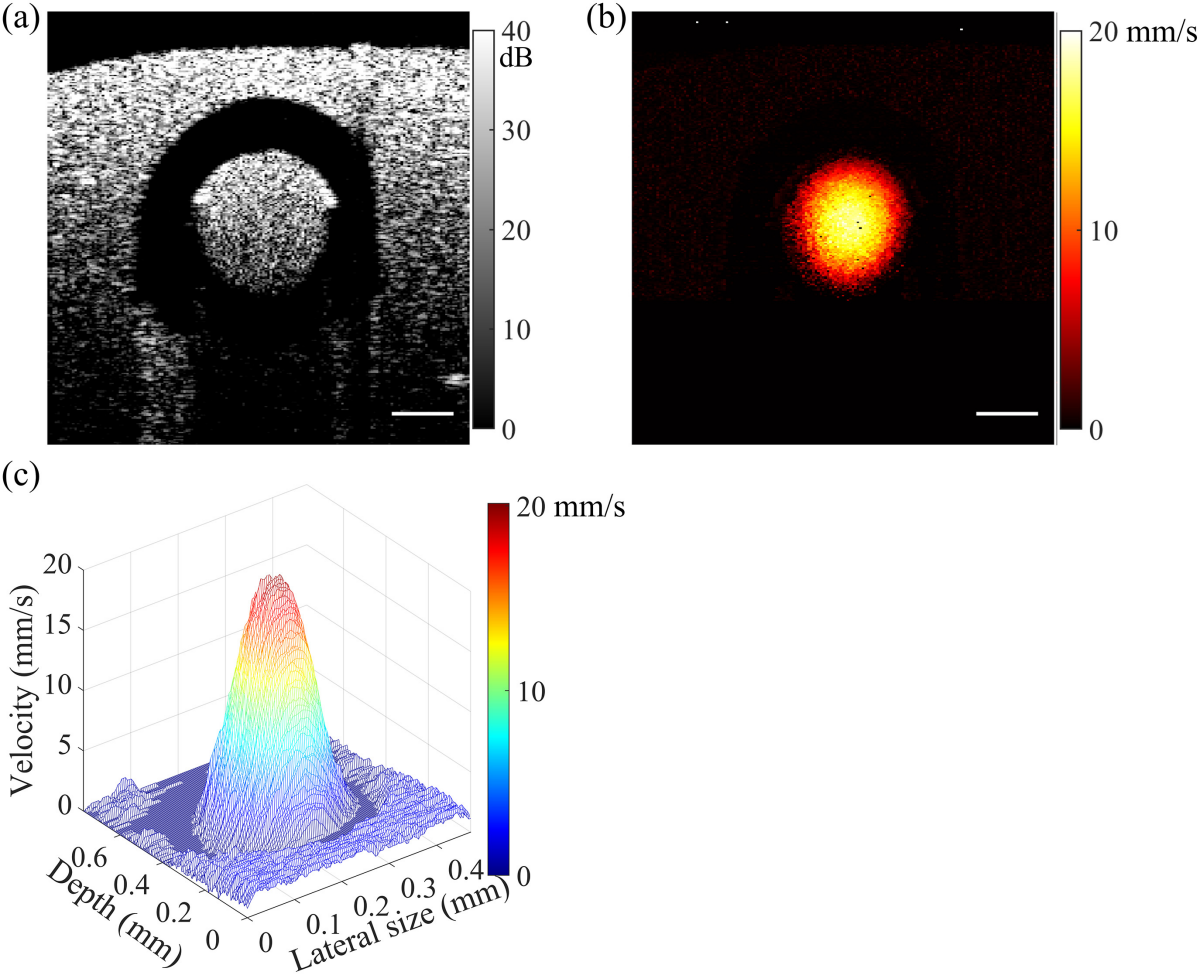
(a) and (b) OCT B-scan and the resulting flow velocity image of the flow phantom, respectively; (c) 3D view of the flow profile of panel (b). Scale bars: 100  μm.

Figure 3 shows the results from a 2D phantom scan. Figure 3(a) shows the cross-sectional OCT B-scan, where the capillary wall shows an extremely low signal (black) due to its transparency. The flow region is located inside the capillary wall while the static silicone matrix is located outside of it. Figure 3(b) shows the flow velocity image generated with enhanced STdOCT from the same area shown in Fig. 3(a). Figure 3(c) provides a 3D perspective of Fig. 3(b), with the flow velocity encoded in both color and height. The laminar flow structure within the capillary is distinctly visible in Fig. 3(c), demonstrating the high resolution and accuracy of this method.

### 3.2 Normal Skin Results

**Fig. 4:**
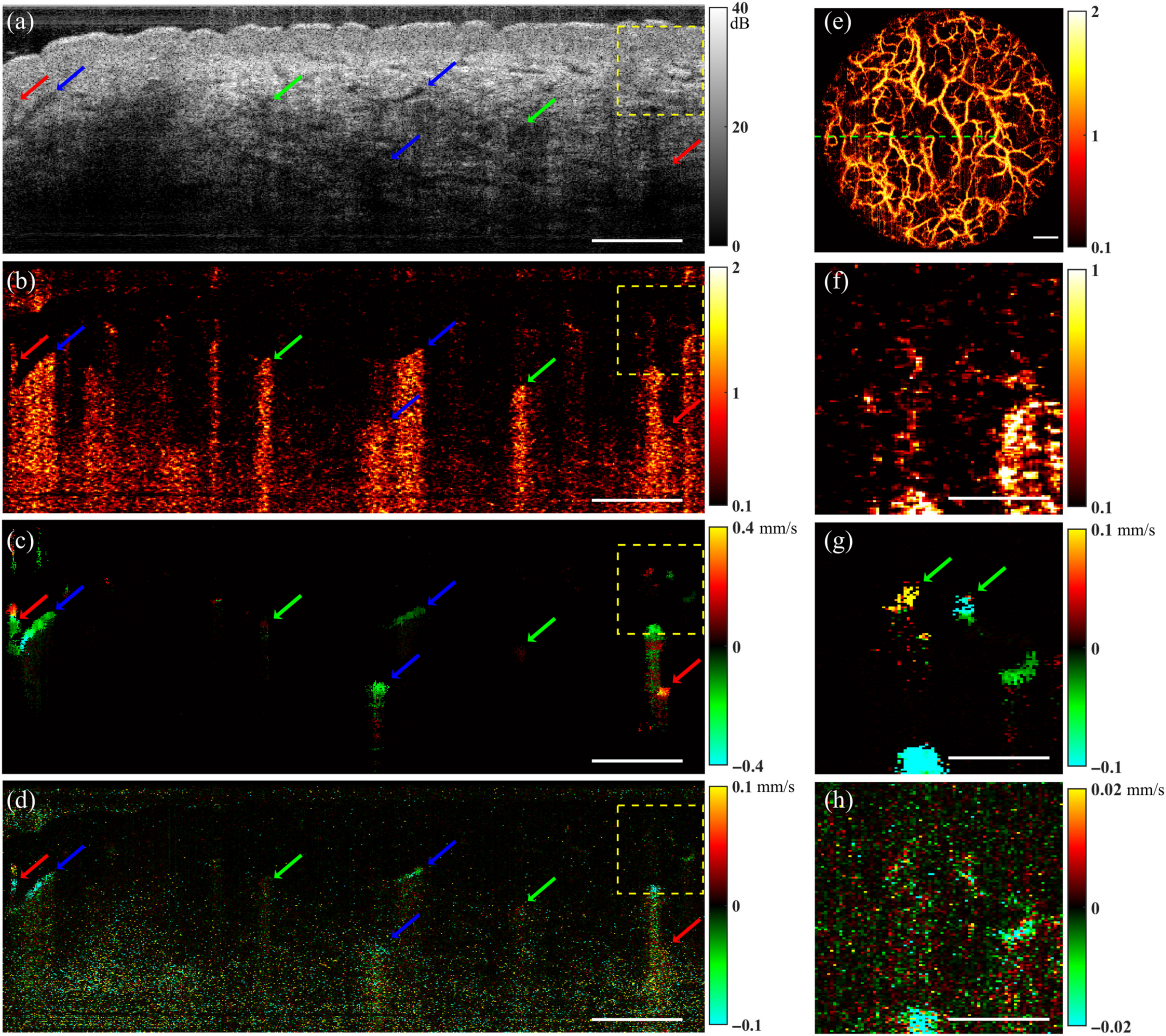
Images of the forearm skin of a healthy subject, *in vivo*. (a)–(d) Cross-sectional images of the OCT signal, speckle decorrelation, enhanced STdOCT, and DOCT, obtained from the location of the green dashed line in panel (e); the blue arrows mark three vessels with shadow artifacts in panel (b); the red arrows mark two vessels located under other vessels, mainly visible in panel (c); the green arrows indicate two vessels that are only visible in panel (b); (e) projection of the blood vessels from skin surface to a depth of 600  μm with a dashed line marking the locations for 2D scanning; (f)–(h) magnified images of the regions outlined in panels (b)–(d), respectively; the green arrows in panel (g) mark two vessels that are not visible in panels (f) and (h). Scale bars in (a)–(e): 500  μm. Scale bars in (f)–(h): 200  μm.

Figure 4 shows flow imaging of the forearm skin of a healthy 29-year-old subject. The projection of the speckle decorrelation image in Fig. 4(e) shows the distribution of the blood vessels from the skin surface to a depth of 600  μm. Within this OCT FOV, a 2D scans for flow measurement was obtained from the location indicated by the green dashed line. Figures 4(a)–4(d) are the OCT, unweighted speckle decorrelation, enhanced STdOCT, and DOCT images from the location at the dashed line in Fig. 4(e), respectively.

Blood vessels are marked with colored arrows to highlight the different signal features. First, the blue arrows mark three blood vessels with strong shadow artifacts in Fig. 4(b), which are almost eliminated by the enhanced STdOCT method in Fig. 4(c). This observation suggests improved immunity to shadow artifacts for enhanced STdOCT.

The red arrows in the right section of Figs. 4(a)–4(d) mark blood vessels, which are confounded by the shadow artifacts induced by an overlying vessel in the speckle decorrelation [Fig. 4(b)]. This vessel is barely visible but can be identified in the projection image of the vessels in Fig. 4(e) at an intersection of three vessels. In contrast, the flow image in Fig. 4(c) clearly shows this vessel, which has a flow direction opposite to the top vessel. Another two blood vessels marked with red arrows on the left in Figs. 4(a)–4(d) demonstrate a similar case of overlapping vessels with opposite flow directions. These cases show that enhanced STdOCT, due to its high immunity to shadow artifacts, enables flow measurement in deep vessels that are overlapped by superficial vessels, which cannot be visualized with speckle decorrelation.

In the speckle decorrelation image [Fig. 4(b)], several vessels are visible, which are marked by green arrows, but they are hardly visible in the enhanced STdOCT image [Fig. 4(c)]. We speculate that these vessels may be perpendicular to the incident beam, leading to a low axial velocity, which results in a low signal in the enhanced STdOCT image.

Figure 4(d) shows the DOCT image as an alternative for measuring blood flow velocity. The axial velocity and direction were also acquired using the DOCT. However, there is significantly more noise than in the enhanced STdOCT image, particularly in the low OCT SNR region. This further demonstrates that the DOCT requires a higher phase stability.

The region outlined by the dashed yellow squares in Figs. 4(b)–4(d) is magnified in Figs. 4(f)–4(h) with the color maps adjusted to enhance the visibility of these vessels for each method. Green arrows mark 2 vessels with a diameter smaller than 50  μm, and they are poorly visible in speckle decorrelation and DOCT images, but clearly visualized with the enhanced STdOCT method, demonstrating the superior performance of the enhanced STdOCT method for imaging small vessels.

The 3D structure of the microvasculature can be clearly seen in the healthy skin image when speckle decorrelation is used. The speckle decorrelation method cannot measure blood flow speed, but it can help in understanding the blood vessel orientation. Both the DOCT and the enhanced STdOCT methods measure the axial velocity within blood vessels; however, the enhanced STdOCT approach clearly outperformed the DOCT in terms of image quality and the capacity to identify small blood vessels, and its ability to quantify flow in the presence of a shadow artifact.

### 3.3 Burn Scar Results

**Fig. 5:**
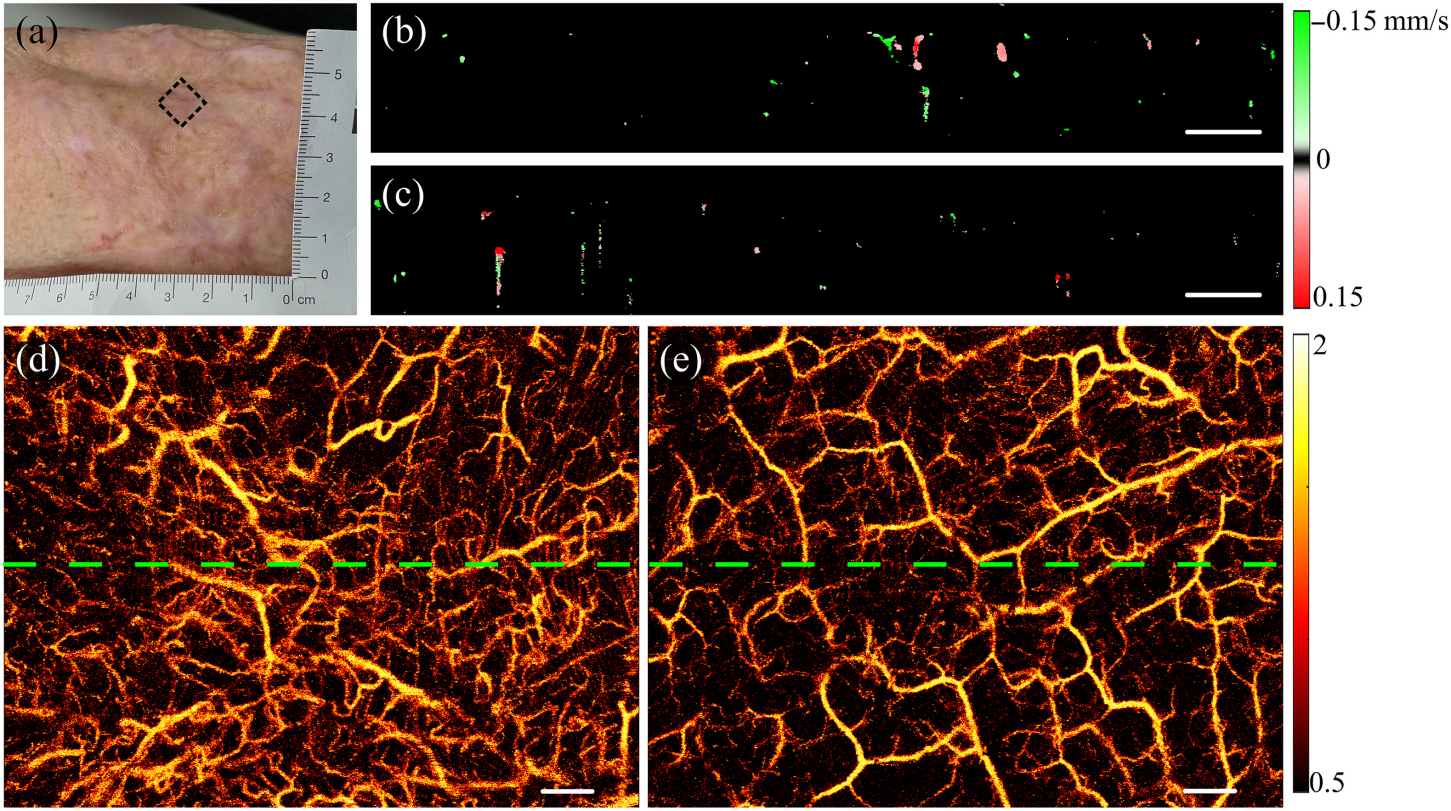
*In vivo* images of a second-degree burn scar on the left forearm. (a) Photograph of the scar with the black dotted box indicating the scan location; (b) and (c) cross-sectional flow images generated by enhanced STdOCT from the scar and adjacent healthy tissue, respectively, as indicated by the green lines in panels (d) and (e); (d) and (e) projection images generated by speckle decorrelation from the burn scar indicated by the black box in panel (a) and adjacent healthy tissue, respectively. Scale bars in (b)–(e): 500  μm.

Figure 5 shows images of second-degree burn scars and healthy tissue on the left forearm of a 58-year-old male. A photograph of the burn scar is shown in Fig. 5(a), where *in vivo* scans were acquired from both the scar, as indicated by the black box in Fig. 5(a), and adjacent healthy tissue. Projection images of the scar and nearby healthy tissue, generated by speckle decorrelation, are shown in Figs. 5(d) and 5(e), respectively. Figure 5(d) shows that the burn scar has a higher blood vessel density, characterized by a greater number of small blood vessels. In addition, large blood vessels within the scar appear more disorganized and tortuous, exhibiting increased curvature. In contrast, large blood vessels in healthy tissue shown in Fig. 5(e) are more extensive.

Cross-sectional flow images from the green lines in Figs. 5(d) and 5(e) are presented in Figs. 5(b) and 5(c), respectively, from the skin surface to a depth of 1 mm. Comparing these images emphasizes that, within the scar area, a number of blood vessels are present in the 0 to 200  μm range, with some exhibiting larger diameters. The majority of vessels in the scar are concentrated in the 0 to 500  μm range. In contrast, healthy tissue demonstrates fewer vessels in the 0 to 200  μm range, with blood vessels evenly distributed from the surface to a depth of 1 mm.

**Fig. 6:**
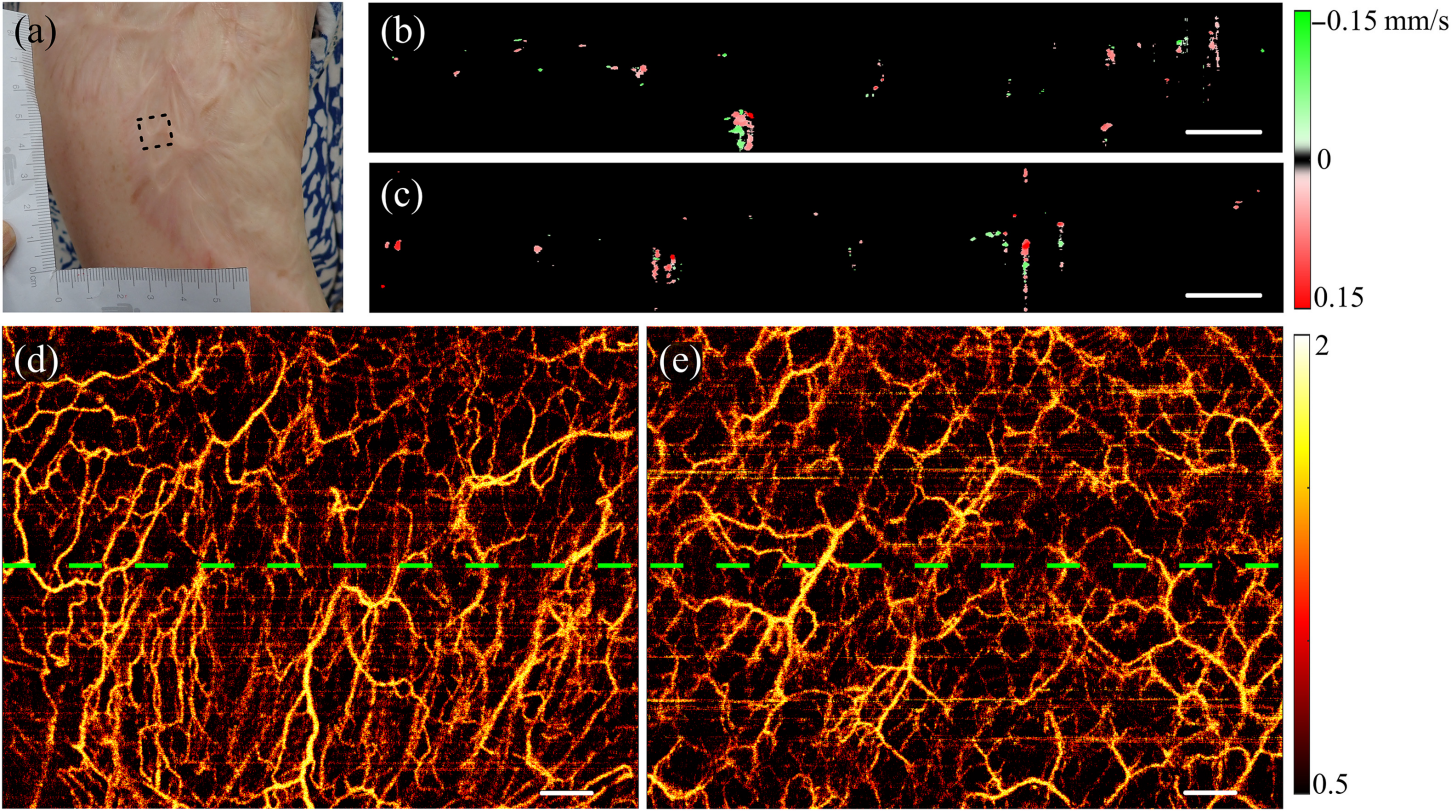
*In vivo* images of a right upper arm with a third-degree burn scar. (a) Photograph of the scar with the black dotted box indicating the scan location; (b) and (c) cross-sectional flow images generated by enhanced STdOCT from the scar and adjacent healthy tissue, respectively, as indicated by the green lines in panels (d) and (e); (d) and (e) projection images generated by speckle decorrelation from the burn scar indicated by the black box in panel (a) and adjacent healthy tissue, respectively. Scale bars in (b)–(e): 500  μm.

Figure 6 shows a series of images from a third-degree burn scar and healthy tissue on the upper right arm of a 48-year-old female. Figure 6(a) shows a photograph of the scar, with data collected from both the scar, as indicated by the black box in Fig. 6(a), and adjacent healthy tissue. The speckle decorrelation method was used to generate projection images of the vessels in the scar and neighboring healthy tissue, as shown in Figs. 6(d) and 6(e). These images show a higher blood vessel density in the burn scar, characterized by a greater number of large blood vessels aligned vertically. In contrast, large blood vessels in healthy tissue appear more evenly oriented.

Figures 6(b) and 6(c) exhibit cross-sectional flow images from the green lines indicated in Figs. 6(d) and 6(e), respectively, from the skin surface to a depth of 1 mm. The images indicate that blood vessels in burn scars are concentrated more closely to the skin surface, and the blood vessel density in the burn scar is higher than that in healthy tissue.

A total of 602 blood vessels were extracted from 2D scans from burn scars (including vessels at scar and scar side of the scar margin) and 361 from healthy tissue (including vessels at healthy tissue and healthy side of the scar margin) using the method described in Sec. 2.3. Since blood vessels were obtained in a cross-sectional image, a density unit of blood vessels was introduced as vessels (v) per square millimeter ($v/{\mathrm{mm}}^{2}$). However, due to the limited imaging depth of the technique, ∼95% of the blood vessels are located within 1 mm of subcutaneous tissue. Therefore, when calculating the density of blood vessels, we excluded vessels that were located beyond a depth of 1 mm. The results show that the density of blood vessels in the burn scar on average was $7.8\;v/{\mathrm{mm}}^{2}$ while it was $6.7\;v/{\mathrm{mm}}^{2}$ in the scar area near the scar edge. The blood vessel density in healthy tissue was $4.4\;v/{\mathrm{mm}}^{2}$ and $4.3\;v/{\mathrm{mm}}^{2}$ in the healthy area near the scar edge, demonstrating a 67% increase in vessel density in the burn scar.

**Fig. 7:**
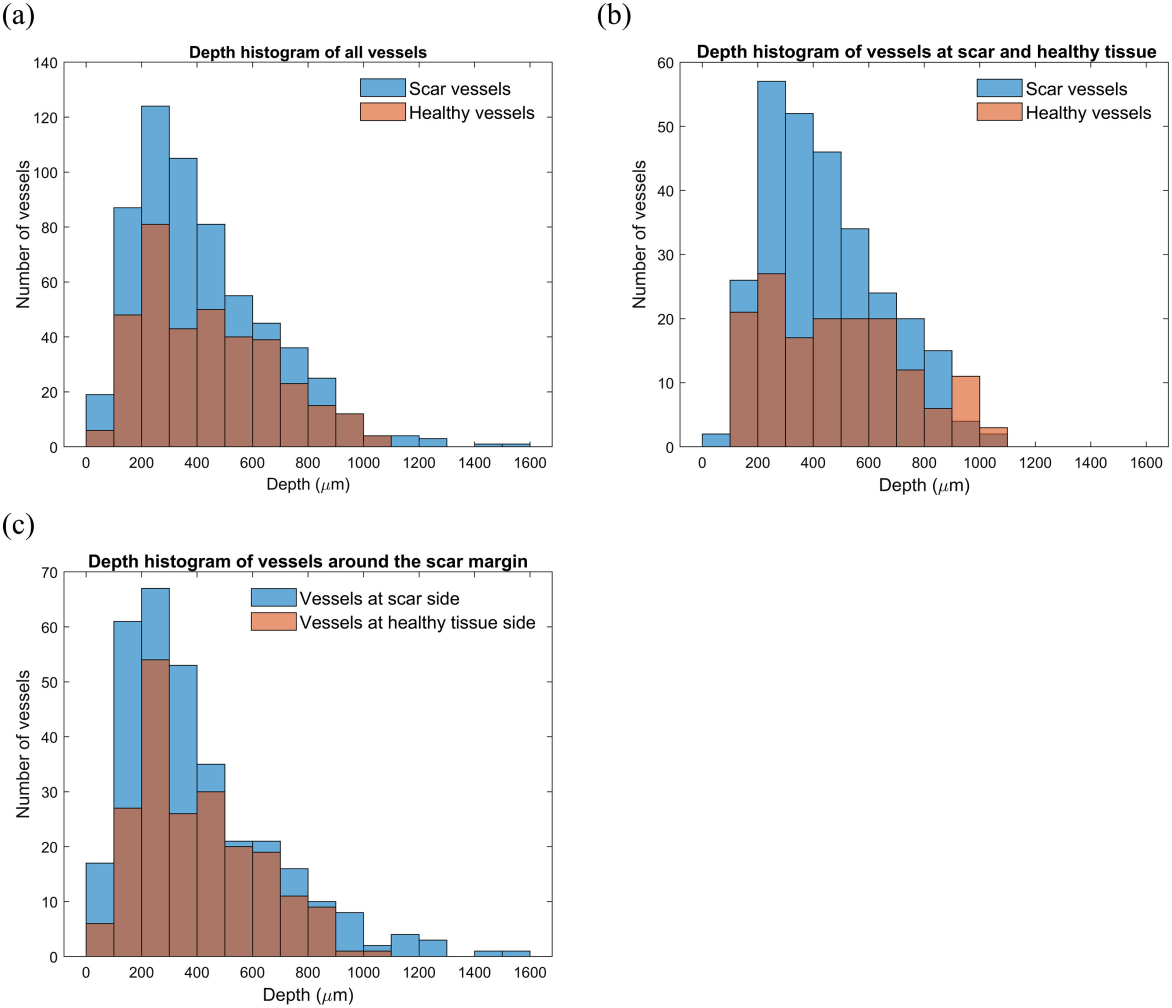
Histograms of blood vessel depth. (a) Distribution of blood vessel depths in the tissue, including vessels within the scar, healthy tissue, and the scar margin; (b) comparison of blood vessel depth distributions in scar and healthy tissue; and (c) blood vessel depth distribution at the scar margin.

Figure 7 shows the distribution of blood vessels as a function of their depth. In Fig. 7(a), the depth information encompasses all blood vessels, including those within the scar, healthy tissue, and the scar margin. The analysis indicates that, in the case of burn scars, most of the blood vessels are concentrated in the superficial layer. As the depth increases, their number significantly reduces. This decrease is attributed to a combination of factors, including the existence of larger blood vessels at deeper depths. As a result, with a consistent cross-sectional area, the presence of these larger vessels contributes to an overall reduction in the number of vessels observed. Moreover, it should be emphasized that this initial decrease is further compounded by the effect of signal attenuation at greater depths. In contrast, healthy tissue shown in Fig. 7(b) displays a relatively uniform distribution of blood vessels throughout the depth, with only a reduction in their number at depths below 700  μm. This decrease is mainly caused by the difficulties created by image signal attenuation at these deeper levels. However, the distribution of blood vessels in the burn scar area was different, with a significantly greater number of vessels observed at a superficial depth of 100 to 600  μm compared to healthy tissue. This observation suggests that scar tissue may exhibit increased superficial vascular growth. Figure 7(c) shows that at the boundary between healthy tissue and burn scar, the healthy tissue exhibits a higher concentration of blood vessels at a depth of 200 to 300  μm. This is possibly due to the presence of blood vessels in the scar that is more densely distributed in the depth range of 100 to 500  μm.

**Fig. 8:**
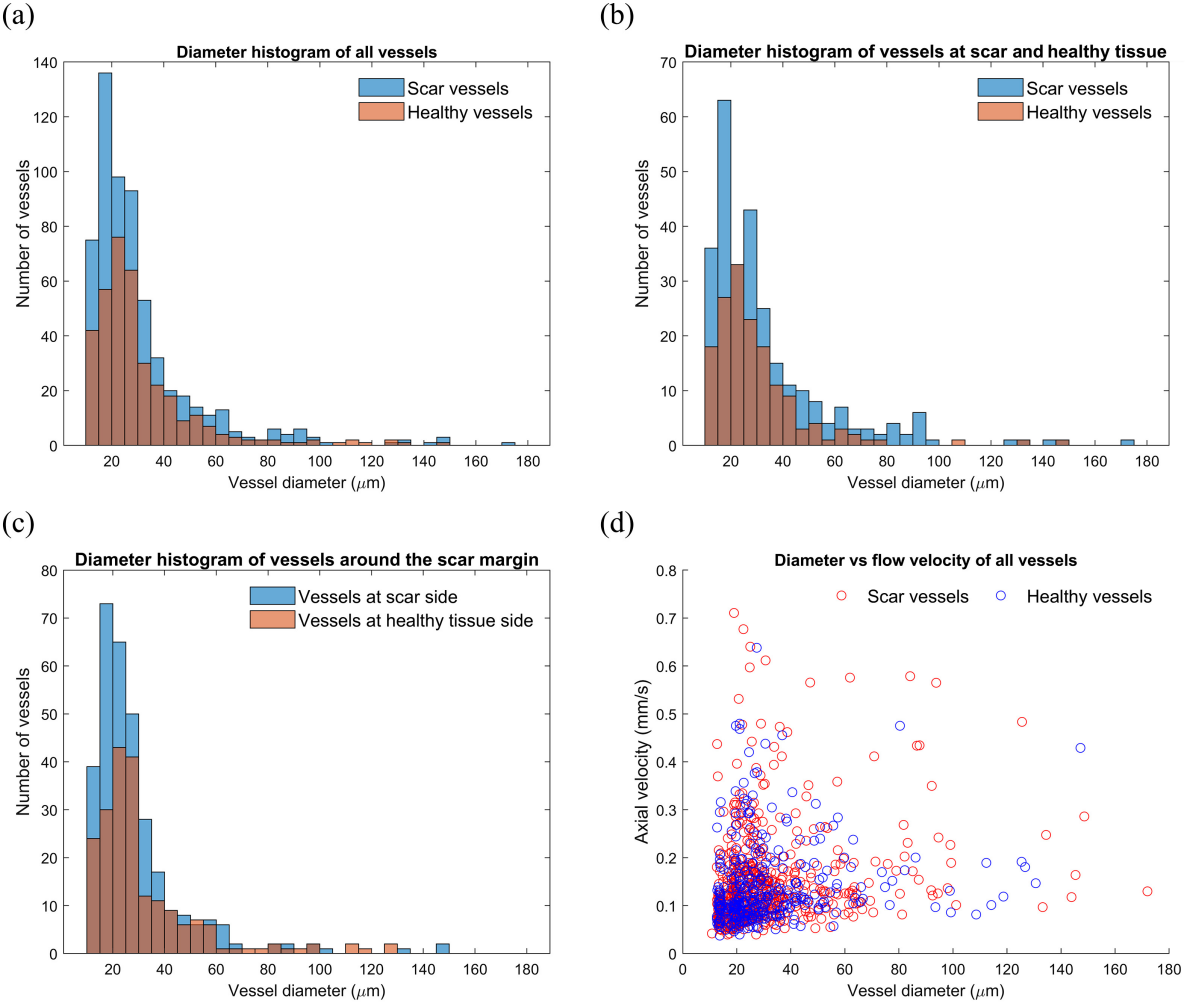
(a) Size distribution of blood vessels in the tissue, including vessels within the scar, healthy tissue, and the scar margin; (b) comparison of blood vessel size distributions in scar and healthy tissue; (c) blood vessel size distribution at the scar margin; and (d) relationship between blood vessel size and flow velocity.

The distribution of vessel sizes is shown in Fig. 8. The histograms in Figs. 8(a) and 8(b) show a dense distribution of blood vessels in healthy tissue, centered at 20 to 25  μm for the blood vessels in healthy tissue. In contrast, the blood vessels in the scar tissue are more concentrated below 30  μm, with a higher density of vessels in the 15 to 20  μm range. Specifically, the number of blood vessels in the 15 to 20  μm range is 130% higher in scars compared to healthy tissue. The scar tissue has more vessels larger than 80  μm while in healthy tissue, vessels larger than 80  μm were rare. In addition, the number of blood vessels larger than 80  μm increased significantly in the healthy tissue at the scar boundary, as seen in Fig. 8(c). It should be noted that the minimum threshold for vessel size was set at 3 pixels in diameter, equivalent to ∼11  μm, to mitigate the impacts of noise. This setting was made based on observations during the blood vessel analysis in the B-scan. Certain signals appearing to be vessels in the B-scan did not always correspond accurately to the actual vessels in projection view. These variations were attributed to noise, often appearing as one or two pixels. Applying this threshold effectively removed the noise artifacts and enabled the clear identification of blood vessels. Figure 8(d) shows the relationship between blood vessel size and flow velocity. As shown in the Fig. 8(d), the scar area exhibits the highest flow velocity across different vessel sizes. This suggests that blood flow velocity in the scar area is faster than in healthy tissue.

**Fig. 9:**
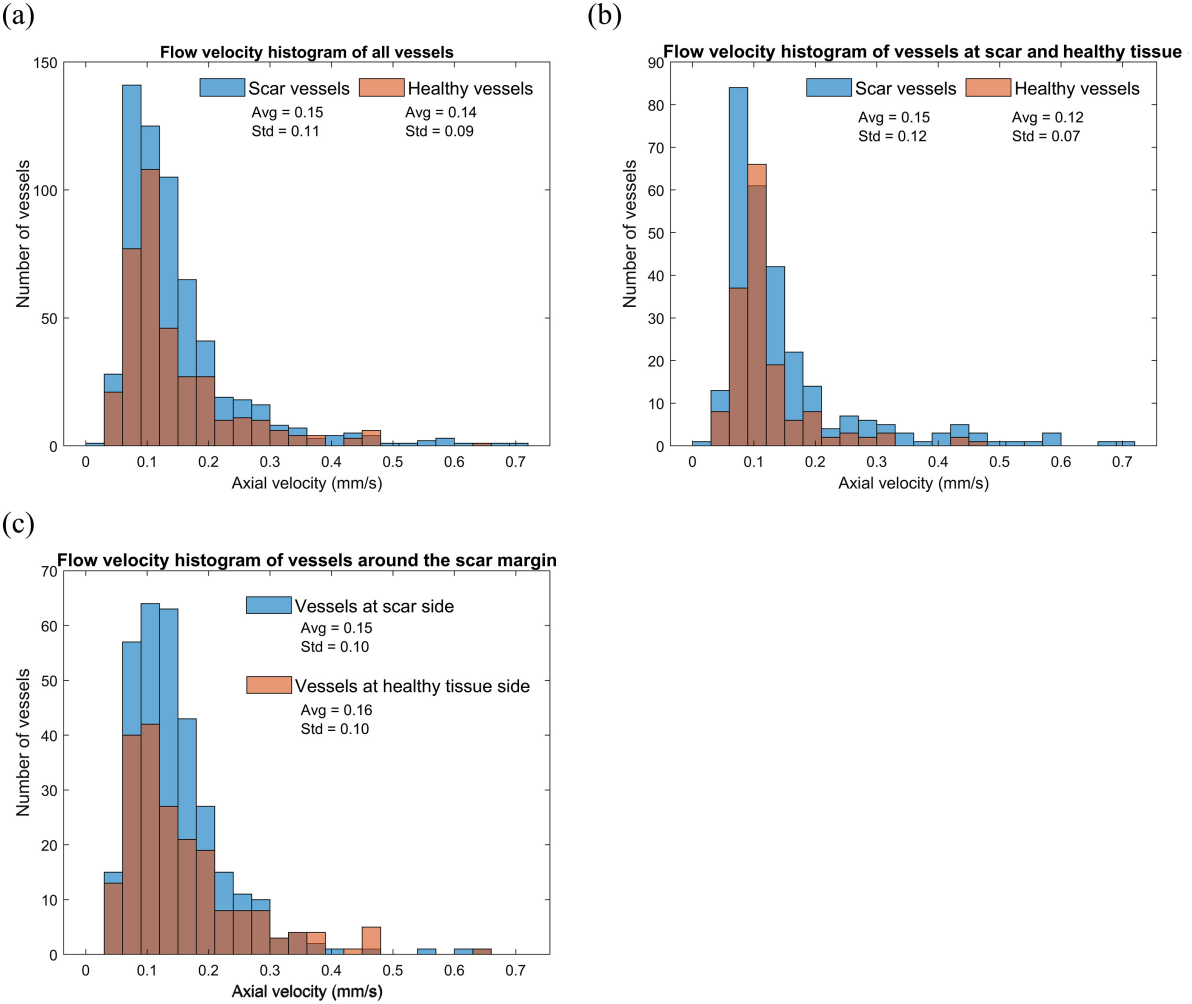
Histograms of blood flow speed. (a) Distribution of blood flow speeds in the tissue, including vessels within the scar, healthy tissue, and the scar margin; (b) comparison of blood flow speed distributions in scar and healthy tissue; and (c) blood flow speed distribution at the scar margin.

Figure 9 shows the distribution of blood vessel flow velocity. Figure 9(a) shows the axial flow velocity of all blood vessels, including those within the scar, healthy tissue, and the scar margin. The analysis reveals that the majority of blood vessels exhibit flow velocities ranging from 0.06 to 0.20  mm/s. The average flow velocity in the scar area was 0.15  mm/s, representing a 7.1% increase compared to the average flow velocity in healthy tissue (0.14  mm/s).

Figure 9(b) shows that the flow velocity in the scar region is 25% higher compared to the healthy tissue area. Notably, the scar region exhibited a number of blood vessels with a flow velocity >0.3  mm/s. In contrast, healthy tissue did not show any blood vessels with a velocity exceeding 0.5  mm/s. These findings suggest that the scar tissue exhibits an increased average flow velocity and increased number of blood vessels with high blood flow velocity.

In Fig. 9(c), we observed that at the boundary, the average velocity in healthy tissue was 6.7% greater than that in scars. This difference can be attributed to the uneven skin surface observed at the scar border, which leads to a more parallel distribution of blood vessels in the measurement direction. As a result, a relatively larger axial flow velocity is observed. These findings indicate that scars have a distinct vascular network that affects the blood flow velocity and distribution in comparison to healthy tissue.

## 4 Discussion

This paper provides a comprehensive description of the theory and computation process for the joint STdOCT method. Supported by the theory, we proposed the enhanced STdOCT method, which improves the flow signal as compared the original joint STdOCT method by integrating additional optimization steps. For example, the enhanced STdOCT method incorporates zero-padding and Gaussian-wrapping techniques to reduce noise interference and increase robustness of the flow signal, and curve-fitting techniques to identify flow peaks. Overall, the enhanced STdOCT method contributes to a method with increased accuracy, reliability, and comprehensiveness.

Although both the enhanced STdOCT and DOCT are restricted to measuring axial flow velocity, they exhibit several distinct features. First, enhanced STdOCT analyzes the flow signature from the complex OCT signal, whereas conventional DOCT only uses the OCT phase signal. This difference may contribute to the observed discrepancies in flow velocity measurements. Second, enhanced STdOCT does not require phase unwrapping, simplifying the data processing. Moreover, the enhanced STdOCT showcases exceptional sensitivity even in low OCT SNR conditions. This feature is valuable in effectively detecting flow within vessels located deep in the skin or affected by shadow artifacts. In comparison to DOCT, the enhanced STdOCT method demonstrated improved performance.

Uribe-Patarroyo et al.^43^ presented a quantitative technique to improve the robustness and noise tolerance of velocity measurements using speckle decorrelation with OCT. Their work provides insightful technical strategies to address the challenges associated with flow velocity determination using speckle decorrelation, but the method has several limitations. First, this speed measurement exhibits an intrinsic non-linearity, particularly when dealing with slow flows. This characteristic introduces the potential for compromised accuracy. As a result, the accuracy of low flow rates, such as in microvascular blood flow measurements, could be adversely affected. Second, the complex data processing and computational steps involved in this technique could be time-consuming. These limitations may preclude the application to burn scar assessment in the clinic.

The most widely used burn scar assessment technique is the VSS, which combines subjective assessment with a semiquantitative methodology.^8^ However, despite its wide use, the VSS has limitations in terms of validity and reliability, particularly when assessing large or irregular scars.^44^ In our study, we aimed to go beyond conventional assessments that focus on macroscopic features such as thickness, color, pigmentation, and roughness as assessed by scar scales. Instead, we looked at more subtle characteristics such as blood vessel flow velocity, size, and depth distribution. Our method provides objective insights, but its application in the clinical settings may be limited by the complexity of the equipment and the need for skilled operators, which could be improved in following work.

Our results showed that burn scars exhibited increased blood vessel density, primarily concentrated in the 15 to 20  μm size range and depths of 100 to 300  μm. These regions also showed increased flow velocities. However, it is important to acknowledge that the unique diversity of burn scars, in type, location, and degree, may result in variations that need to be investigated in detail. Furthermore, blood flow speed in vessels changes over time during a heartbeat, resulting in varying flow velocity of individual vessels at different times. In addition, the flow velocities in blood vessels of different sizes can differ significantly. As such, examining the flow velocity of a single vessel at a single time may not provide a meaningful representation of the overall flow in the area. Collectively, our study highlights the potential value of investigating nuanced characteristics of burn scars. However, further research is needed to validate our findings and improve our understanding of burn scars.

The distribution of blood vessels in healthy tissue and corresponding scar tissue from the same individuals was significantly different, as seen in Figs. 5 and 6. Blood vessels are distributed densely in scar tissue, with large blood vessels displaying more tortuous connectivity and a less uniform distribution, as shown in Fig. 5(d). In addition, large vessels may appear to converge in a specific direction, as shown in Fig. 6(d). In contrast, healthy tissue presents a uniform pattern, characterized by larger vessels that are evenly distributed throughout the tissue. The contrast between the uniform distribution of vessels observed in healthy skin tissue and the converging and less uniform distribution observed in scar likely reflects the result of healing and the requirement for angiogenesis to sustain the newly formed tissue in a short period of time in contrast to the development of vasculature of the normal skin. However, further work to understand the genesis of these observed differences is required.

This study investigated the vascular distribution, flow velocity, vessel density, vessel depth, and vessel size of burn scars in comparison to healthy skin. The vascular characteristics may also vary among scars depending on the type of burns. However, the study population in this work only comprised 9 scar patients, mostly caused by open flame burns (n=5), and several by electric shock, contact and laser burns (n=1 and 2 for each). This small study population precluded a comparison of the measured vascular characteristics among the burn types. Further research could seek to provide such a comparison among burn types by collecting data from a much larger study population.

In the study by Liew et al.,^28^ OCT was employed to analyze burn scars as well, leading to two key conclusions. First, burn scar demonstrated a larger blood vessel density, with an average area density of 38% compared to 22% for normal skin. Our study supports this finding, showing that the blood vessel density of burn scars is 67% higher than healthy tissue. However, our approach differed as we determined blood vessel density by counting individual vessels within a specific area instead of determining the proportion of blood vessels in the projection map. Second, Liew et al. found that the average of the median diameter of blood vessels in burn scar was 34  μm, in contrast to 23  μm in normal skin, suggesting a higher number of smaller vessels in healthy tissue compared to burn scars. Our findings differ regarding blood vessel size, as our data suggest a higher number of small blood vessels in burn scars compared to healthy tissue. This difference may arise due to three potential reasons. First, our system has a higher axial resolution (5.5  μm in air) compared to Liew’s system (17  μm in air), enabling the detection of smaller blood vessels. Second, we analyzed mature scars, whereas Liew’s study focused on fresher scars, which could lead to differences in blood vessel diameter between the two studies. Finally, our measurement method varied as we determined blood vessel diameter using cross-sectional images while Liew et al. calculated blood vessel diameters with projection images.

The projection images of vessels in Figs. 6(d) and 6(e) showed more artifacts (i.e., horizontal lines) than those in Fig. 5, due to motion between repeated OCT B-scans. This might be due to the different body locations for imaging as Fig. 6 was from the upper arm which was more susceptible to motion due to breathing and heartbeat than the forearm in Fig. 5. To minimize the motion artifacts, patients were instructed to maintain stable during data acquisition, but different patients may vary and lead to the increased residual motion in Fig. 6 from 3D imaging. Overall, compared to previous studies, the motion artifacts in our images were relatively minor.^45^^,^^46^ In addition, it should be noted that quantification of the vessel parameters was performed using the 2D scans, which had a much smaller time interval between repeated A-scans and consequently was not compromised by such residual tissue motion.

This study did not obtain 3D velocity data due to limitations of the commercial system, which cannot apply custom scan patterns and lacks a much higher acquisition speed suitable for optimal detection. However, OCT systems with much higher scanning speeds^47^ may allow for imaging vessel flow velocity in 3D with the enhanced STdOCT method. Obtaining the 3D flow data would enable a more accurate reflection of the differences between burn scars and healthy tissue and provide more precise results for burn scar assessment. Therefore, understanding velocity patterns within burn scars can provide valuable insights for scar assessment, treatment planning, and monitoring of scar progression.

## 5 Conclusion

We have proposed an enhanced STdOCT method with a theoretical explanation for axial flow velocity and direction measurements and demonstrated its performance on both a flow phantom and human skin, *in vivo*. The *in vivo* measurements demonstrated the feasibility and potential of enhanced STdOCT for non-invasive assessment of blood vessels in burn scar patients. The precise measurement of axial flow velocity and flow direction provides comprehensive information about vessel size and depth in burn scars. Our findings indicate that burn scar tissue exhibits higher blood vessel density, altered vessel structures, and increased blood flow velocities compared to healthy tissue. Further data collection is necessary to validate these observations. However, the identified features hold promise for future evaluations and assessments of burn scars. Advancements in high-speed OCT technology would enable real-time 3D imaging, providing clinicians with detailed information about cutaneous microcirculation. This enhanced imaging capability can contribute to more accurate assessments of burn scars. Scar treatment strategies, such as pressure and laser therapies, have the potential to modulate the size and flow characteristics of blood vessels. This technique offers an opportunity to observe and evaluate the effectiveness of these treatment interventions. In conclusion, this method shows promise in the assessment of burn scars and has the potential to be used in clinical settings.

## Data Availability

The data supporting the findings of this study are available upon reasonable request. Requests for data access should be directed to Qiang Wang. Access to the data is subject to any necessary permissions or ethical considerations.
